# Navigating the gut-bone axis: The pivotal role of Coprococcus3 in osteoporosis prevention through Mendelian randomization

**DOI:** 10.1097/MD.0000000000038861

**Published:** 2024-07-19

**Authors:** Jun Ma, Xin-Ran Wang, Yu-Xin Zhou, Wei-Jin Zhou, Jian-Nan Zhang, Chong-Yi Sun

**Affiliations:** a Department of Orthopedics, 2nd Hospital of Mudanjiang People, Heilongjiang Province, China; b Department of Critical Care Medicine, The First Affiliated Hospital of Harbin Medical University, Heilongjiang Province, China; c Department of Orthopedics, The Second Affiliated Hospital of Harbin Medical University, Heilongjiang Province, China.

**Keywords:** Coprococcus3, gut microbiota, mendelian randomization, osteoporosis

## Abstract

Osteoporosis (OP) constitutes a notable public health concern that significantly impacts the skeletal health of the global aging population. Its prevalence is steadily escalating, yet the intricacies of its diagnosis and treatment remain challenging. Recent investigations have illuminated a profound interlink between gut microbiota (GM) and bone metabolism, thereby opening new avenues for probing the causal relationship between GM and OP. Employing Mendelian randomization (MR) as the investigative tool, this study delves into the causal rapport between 211 varieties of GM and OP. The data are culled from genome-wide association studies (GWAS) conducted by the MiBioGen consortium, in tandem with OP genetic data gleaned from the UK Biobank, BioBank Japan Project, and the FinnGen database. A comprehensive repertoire of statistical methodologies, encompassing inverse-variance weighting, weighted median, Simple mode, Weighted mode, and MR-Egger regression techniques, was adroitly harnessed for meticulous analysis. The discernment emerged that the genus Coprococcus3 is inversely associated with OP, potentially serving as a deterrent against its onset. Additionally, 21 other gut microbial species exhibited a positive correlation with OP, potentially accentuating its proclivity and progression. Subsequent to rigorous scrutiny via heterogeneity and sensitivity analyses, these findings corroborate the causal nexus between GM and OP. Facilitated by MR, this study successfully elucidates the causal underpinning binding GM and OP, thereby endowing invaluable insights for deeper exploration into the pivotal role of GM in the pathogenesis of OP.

## 1. Introduction

Osteoporosis (OP) has emerged as a paramount concern in public health, gravely compromising the skeletal well-being of the global aging populace.^[1]^ It has significantly escalated socio-economic burdens among the elderly worldwide, while simultaneously compromising patient welfare. The escalating prevalence of OP, attributed to societal aging and shifts in dietary habits, underscores its profound detriment.^[2]^ While preventative and therapeutic strategies have developed,^[3]^ a vast majority of patients still lack timely and efficacious diagnosis and treatment. Recent observational studies on OP, through comprehensive genome-wide association studies (GWAS) and meta-analyses, have elucidated the role of the G-protein-coupled receptor, GPR43. This receptor, via the GPR43-βarr2 signaling pathway, suppresses NF-kB activity,^[4–6]^ thus modulating bone metabolism. These revelations provide novel perspectives on the aetiology of OP from both genetic and immunological standpoints.

The human gastrointestinal tract harbours myriad symbiotic microbial communities, numbering in the trillions,^[7]^ crucial for maintaining our health. With the relentless progress in high-throughput sequencing technologies, mounting evidence underscores the pivotal role of gut microbiota (GM) in bone metabolic processes.^[8]^ The dynamic fluctuations of these microbial communities are intricately linked to the preservation of bone mass and quality.^[9]^ Certain observational meta-analyses suggest a correlation between these microbial shifts and OP, with notable regional disparities in dominant microbial populations.^[10]^ Research affirms that GM can influence bone metabolism through a multitude of mechanisms. These mechanisms are interwoven with various modifiable factors, such as gut metabolic byproducts, immune functionality, intestinal epithelial barrier functions, nutrient absorption metabolism, estrogens, and endocrinology.^[11,12]^

Mendelian randomization (MR) is a robust technique for deducing causal relationships, utilizing genetic variations as instrumental variables (IV) to investigate the causal effects of exposures on outcomes. In this context, we employ GM as the exposure and OP as the outcome, utilizing MR analysis to explore the causal nexus between GM and OP.

## 2. Methods

### 2.1. Study design

**Figure 1.**
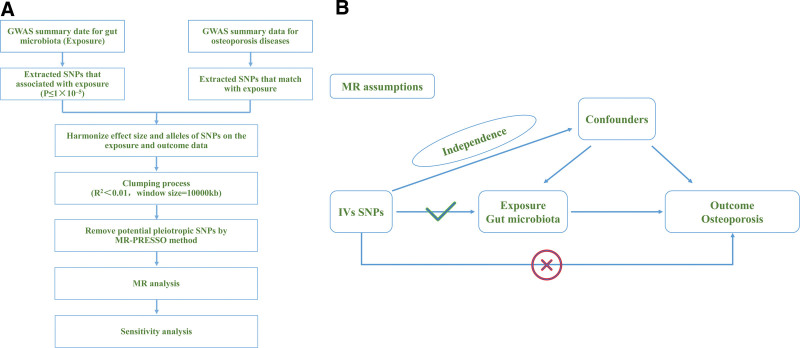
(A) Flowchart of the MR Study (B) Three assumptions of Mendelian randomization. GWAS = genome-wide association studies, IV = instrumental variable, IVW = inverse-variance weighted, MR = Mendelian randomization, SNP = single nucleotide polymorphism, UKB = UK Biobank, WM = weighted median.

The central focus of investigation lies in the 211 gut microbial species under scrutiny, while OP is defined as the outcome of study (see Fig. 1). A preliminary screening was carried out to identify gut microbial species significantly linked with OP, thus paving the way for an extensive MR analysis. This analysis is built upon 3 fundamental assumptions: IVs exhibit an association with the exposure, IVs remain independent from any confounding factors, and IVs impact the outcome solely through the conduit of the exposure pathway.^[13]^

### 2.2. Data sources

The genetic data pertaining to gut microbial communities is sourced from the latest Genome-Wide Association Study (GWAS) summary data by the MiBioGen consortium, encompassing a cohort of 18,340 individuals across 24 cohorts. We assessed 211 gut microbial communities spanning diverse taxonomic tiers, which include phylum, class, order, family, and genus.^[14]^ This study scrutinized the composition of the GM, based on 3 distinct variable regions (V1-V2, V3-V4, and V4) in 16S rRNA sequencing. Moreover, it employed mapping of microbial quantitative trait loci (mbQTL) to pinpoint genetic variants that influence the relative abundance of microbial taxa.^[14]^ The genetic information for OP comprises 7547 cases and 455,386 controls from the UK Biobank, 7788 cases and 204,665 controls from BioBank Japan, and 3203 cases and 209,575 controls from the FinnGen database. The GWAS summary data is drawn from databases with European ancestry, ensuring minimal likelihood of sample overlap. Details of exposures and outcomes are presented in Table 1. The foundational studies underpinning this data have received endorsement from the pertinent ethical review committees.^[15,16]^ As a result, this study does not necessitate supplementary ethical clearance.

**Table 1:** Details of the exposure and outcome.

Dataset	Traits	Consortium or study	Sample size	Populations
Exposure 1	211 GM taxa	PopGen/FoCus	1812 individuals	European
Outcome 1	Osteoporosis	UK biobank	7547 cases and 455,386 controls	European
Outcome 2	Osteoporosis	Fin biobank	3203 cases and 209,575 controls	European
Outcome 3	Osteoporosis	BBJ consortium	7788 cases and 204,665 controls	East Asian

GM = gut microbiome.

### 2.3. Selection of IV

The criteria guiding the selection of IVs encompass: (1) Preference for Single Nucleotide Polymorphisms (SNPs) associated with each genus at a genome-wide significance level (*P* < 1.0 × 10^–5^)^[17]^; (2) Utilization of European sample data from the 1000 Genomes Project as a reference panel to compute linkage disequilibrium (LD) among SNPs, retaining only the SNP with the lowest *P* value among those with R^2^ < 0.001 (clustering window size = 10,000 kb); Exclusion of SNPs with a minor allele frequency ≤ 0.01; In cases of palindromic SNPs, deducing the forward strand allele using allele frequency information.

### 2.4. Statistical analysis

**Figure 2.**
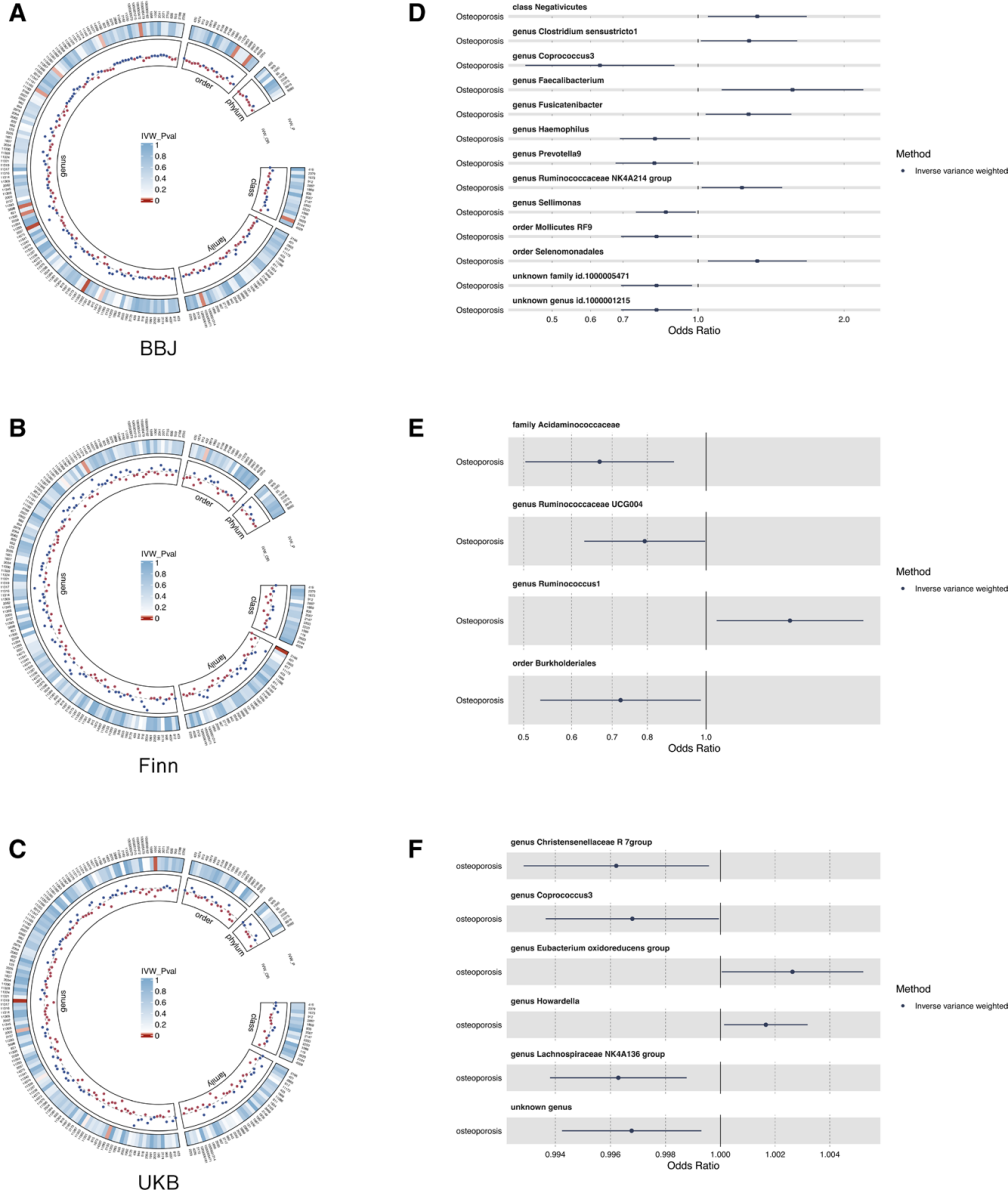
Analysis of causal relationships between gut microbiota (GM) and osteoporosis (OP). Panels A, B, and C illustrate the genome-wide association analysis between GM and OP (*P* < 1 × 10^−5^). Panels D, E, and F present the MR results of GM communities with a causative link to OP.

This study employs a diverse range of methods, including Inverse-Variance Weighted (IVW), Weighted Median, Simple Mode, Weighted Mode, and MR-Egger regression techniques, to elucidate potential causal relationships between GM and OP. The IVW method amalgamates meta-analysis techniques with estimates from each SNP to derive an overarching assessment of the impact of GM on OP. Under the absence of horizontal pleiotropy, IVW results remain unbiased.^[18]^ MR-Egger regression operates under the assumption of instrument strength being independent of direct effects, allowing the intercept term to assess the presence of pleiotropy. A zero intercept signifies a lack of horizontal pleiotropy, as depicted in Figure 2. The findings from the MR-Egger regression align with IVW results.^[19]^ The Weighted Median method can accurately estimate causal associations even when up to 50% of IVs are invalid.^[20]^ The Weighted Mode estimation demonstrates superior capability in detecting causal effects, with reduced bias and a lower Type I error rate compared to MR-Egger regression, especially when the direct effects assumption is violated.^[20]^ MR-PRESSO analysis identifies and seeks to mitigate horizontal pleiotropy by removing significant outliers. Cochran IVW Q statistic gauges the heterogeneity of IVs. To identify potential heterogeneous SNPs, a “Leave-one-out” analysis is performed by sequentially excluding each IV SNP.^[21]^

By employing the q-value package for false discovery rate (FDR) correction, the threshold for the error rate q-value is set at 0.1. Significance is attributed to GM and OP when *P* < .05 and q < 0.1. Conversely, when *P* < .05 and q ≥ 0.1, the relationship between GM and OP is considered to be suggestively associated.^[22]^ All statistical analyses were carried out using R software version 4.2.1 (R Foundation for Statistical Computing, Vienna, Austria). MR analyses were conducted using the TwosampleMR (version 0.5.6)^[23]^ and MR-PRESSO (version 1.0)^[24]^ R packages.

## 3. Results

### 3.1. IV selection and preliminary MR analysis outcomes

Following the application of LD effect and palindromic quality control procedures, a total of 2249 SNPs, as identified by the MiBioGen consortium, emerged as IVs linked with the 211 microbial species DR (*P* < 1 × 10^−5^). These microbial communities encompass 9 phyla (102 SNPs), 16 classes (178 SNPs), 20 orders (215 SNPs), 35 families (382 SNPs), and 131 genera (1372 SNPs). By applying a filter based on an IVW *P* value < .05 and an MR-PRESSO *P* value > .05, all initial positive results from the MR analysis are elaborated in Figure 2.

### 3.2. Causal impact of GM on OP

**Figure 3.**
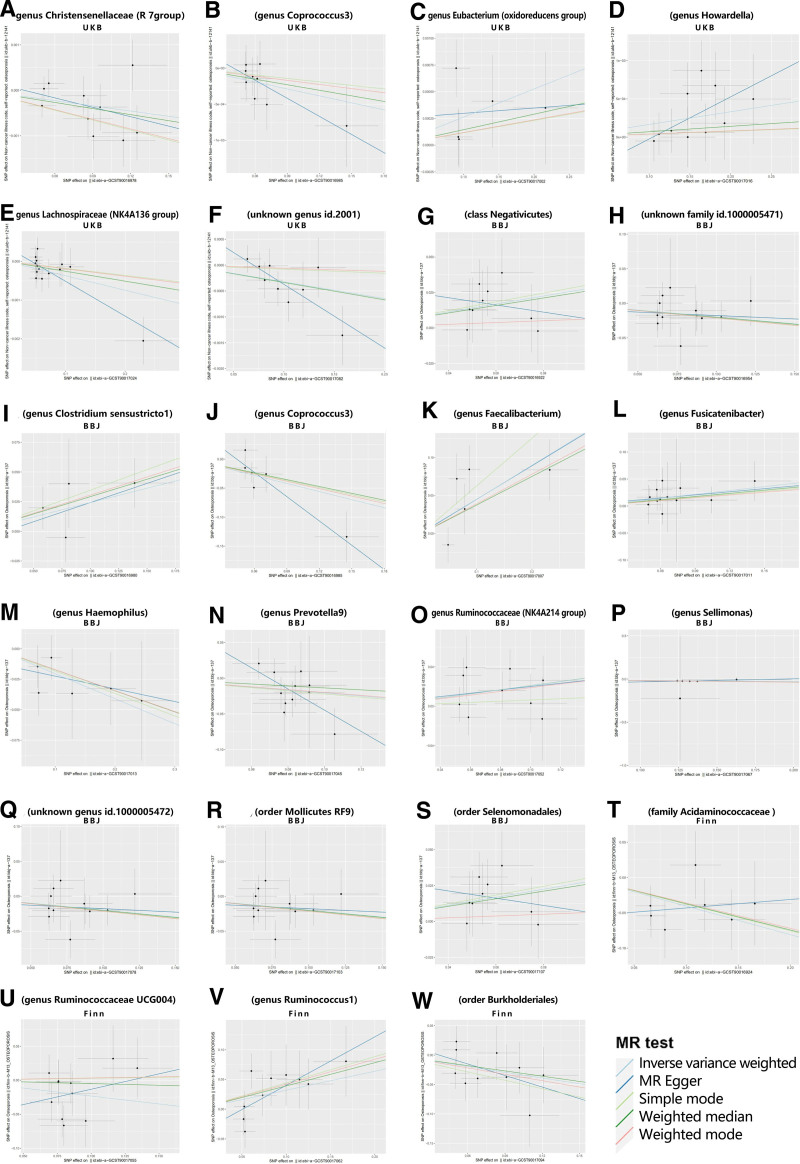
Scatterplot illustrating the causal relationship between gut microbiota and osteoporosis. Note: The subscripts exhibit scatterplots of taxon-SNP associations (x-axis) versus OP-SNP associations (y-axis), with horizontal and vertical lines representing the 95% confidence intervals for each association. The primary MR analysis was executed using the inverse-variance weighted method, followed by tests employing MR-Egger, weighted median, and other techniques. Lines sloping upwards from left to right denote a positive correlation with OP, suggesting a pathogenic causal influence. Downward sloping lines indicate a protective causal effect. MR = Mendelian randomization, SNP = single nucleotide polymorphism.

Evidence from IVW analysis using the UK Biobank (UKB) database indicates an inverse correlation between the genus Coprococcus3 and OP (β = −0.466, SE = 0.180, *P* = .01). MR-Egger results exhibit (β = −1.397, SE = 0.491, *P* = .04). Weighted Median analysis reveals (β = −0.393, SE = 0.206, *P* = .05). Simple Mode demonstrates (β = −0.408, SE = 0.337, *P* = .28). Weighted Mode showcases (β = −0.424, SE = 0.281, *P* = .19). IVW evidence from the BioBank Japan (BBJ) database suggests that the genus Coprococcus3 is inversely related to OP (β = −0.003, SE = 0.001, *P* = .04). MR-Egger results indicate (β = −0.008, SE = 0.006, *P* = .21). Weighted Median results demonstrate (β = −0.002, SE = 0.002, *P* = .20). Simple Mode presents (β = −0.001, SE = 0.003, *P* = .63). Weighted Mode shows (β = −0.001, SE = 0.003, *P* = .57). Comprehensive findings are available in Tables 2, 3, 4, and Figure 3. MR statistical outcomes suggest a protective effect against OP, reducing the risk of its onset.

**Table 2:** MR analysis results of gut microbiota and osteoporosis risk in the UKB database.

	Classification	Nsnp	SE	*P* value	Q-value	OR (95% CI)	Pleiotropy	SE	*P* value	Heterogeneity	
							Egger intercept			Q	*P* value
UKB											
Genus	Christensenellaceae R 7group	10	0.0017	.027	1.939	0.996 (0.9928–0.9995)	0.0002	0.0004	.555	10.056	.345
	Coprococcus3	9	0.0016	.046	1.623	0.996 (0.9936–0.9999)	0.0003	0.0003	.409	3.191	.921
	Eubacterium oxidoreducens group	5	0.0013	.046	1.938	1.002 (1.0000–1.0052)	0.0002	0.0005	.673	4.423	.351
	Howardella	10	0.0007	.032	1.724	1.001 (1.0001–1.0031)	−0.0005	0.0005	.289	5.751	.764
	Lachnospiraceae NK4A136 group	14	0.0012	.003	0.725	0.996 (0.9938–0.9987)	0.0003	0.0002	.086	11.362	.581
	unknown genus	9	0.0012	.012	1.326	0.996 (0.9942–0.9993)	0.0008	0.0004	.111	6.708	.568

MR-PRESSO = Mendelian randomization pleiotropy residual sum and outlier, OR = odds ratio, Q = Cochran Q, SNP = single nucleotide polymorphism.

**Table 3:** MR analysis results of gut microbiota and osteoporosis risk in the Finn database.

	Classification	Nsnp	SE	*P* value	Q-value	OR (95% CI)	Pleiotropy	SE	*P* value	Heterogeneity	
							Egger intercept			Q	*P* value
Finn											
Family	Acidaminococcaceae	7	0.1441	.005	1.055	0.667 (0.5032–0.8852)	−0.0051	0.0432	.258	4.066	.667
Genus	RuminococcaceaeUCG004	11	0.1171	.046	2.455	0.792 (0.6295–0.9966)	−0.0584	0.0541	.308	10.492	.398
	Ruminococcus1	10	0.1422	.025	2.628	1.375 (1.0407–1.8175)	−0.0415	0.0293	.194	8.961	.441
Order	Burkholderiales	10	0.1555	.036	2.579	0.722 (0.5327–0.9803)	0.0242	0.0342	.499	6.862	.651

MR-PRESSO = Mendelian randomization pleiotropy residual sum and outlier, OR = odds ratio, Q = Cochran Q, SNP = single nucleotide polymorphism.

**Table 4:** MR analysis results of gut microbiota and osteoporosis risk in the BBJ database.

Classification		Nsnp	SE	*P* value	Q-value	OR (95% CI)	Pleiotropy	SE	*P* value	Heterogeneity	
							Egger intercept			Q	*P* value
BBJ											
Class	Negativicutes	9	0.1197	.018	0.996	1.324 (1.0472–1.6745)	0.0314	0.0417	.476	4.018	.855
	unknown family	12	0.0858	.021	0.644	0.820 (0.6935–0.9712)	−0.0078	0.0321	.812	6.818	.813
Genus	Clostridium sensustricto1	4	0.1166	.038	0.614	1.273 (1.0134–1.6007)	−0.0081	0.0312	.821	1.889	.595
	Coprococcus3	6	0.1807	.009	1.042	0.627 (0.4402–0.8942)	0.0612	0.0309	.118	8.303	.141
	Faecalibacterium	5	0.1717	.009	1.912	1.565 (1.1177–2.1912)	0.0008	0.0368	.983	6.424	.169
	Fusicatenibacter	12	0.1038	.021	0.738	1.270 (1.0366–1.5575)	0.0033	0.0235	.888	5.674	.894
	Haemophilus	6	0.0851	.016	1.133	0.814 (0.6894–0.9628)	−0.0121	0.0221	.612	1.933	.858
	Prevotella9	11	0.0938	.026	0.567	0.812 (0.6760–0.9766)	0.1034	0.0626	.133	8.939	.537
	Ruminococcaceae NK4A214 group	9	0.0973	.032	0.623	1.231 (1.0173–1.4902)	0.0029	0.0277	.918	6.003	.646
	Sellimonas	6	0.0724	.034	0.598	0.857 (0.7441–0.9886)	−0.0678	0.1113	.575	0.611	.987
	Unknown genus	12	0.0858	.021	0.564	0.820 (0.6935–0.9712)	−0.0078	0.0321	.812	6.818	.813
Order	Selenomonadales	9	0.1197	.018	0.797	1.324 (1.0472–1.6745)	0.0314	0.0417	.476	4.018	.855
	Mollicutes RF9	12	0.0858	.021	0.501	0.820 (0.6935–0.9712)	−0.0078	0.0321	.812	6.818	.813

MR-PRESSO = Mendelian randomization pleiotropy residual sum and outlier, OR = odds ratio, Q = Cochran, SNP = single nucleotide polymorphism.

Analysis of 21 additional GM species reveals a positive correlation with OP. IVW evidence from the UKB database indicates causal links between OP and the following genera: Christensenellaceae R7 group (OR = 0.996, 95%CI: 0.992–0.999, *P* = .027, q = 0.345), Eubacterium oxidoreducens group (OR = 1.002, 95%CI: 1.000–1.005, *P* = .046, q = 0.351), Howardella (OR = 1.001, 95%CI: 1.000–1.003, *P* = .032, q = 0.764), Lachnospiraceae NK4A136 group (OR = 0.996, 95%CI: 0.993–0.998, *P* = .003, q = 0.580), and an unidentified genus (OR = 0.996, 95%CI: 0.994–0.999, *P* = .012, q = 0.568).

Causal relationships between OP and the Acidaminococcaceae family (OR = 0.667, 95%CI: 0.503–0.885, *P* = .005, q = 0.667), as well as the Ruminococcaceae UCG004 genus (OR = 0.792, 95%CI: 0.629–0.996, *P* = .046, q = 0.398), Ruminococcus1 genus (OR = 1.375, 95%CI: 1.040–1.817, *P* = .025, q = 0.440), and Burkholderiales order (OR = 0.722, 95%CI: 0.532–0.980, *P* = .036, q = 0.651) were identified from the Finn database.

Furthermore, from the BBJ database, causal relationships were established between OP and the Negativicutes class (OR = 1.324, 95%CI: 1.047–1.674, *P* = .018, q = 0.855), along with several genera, including Clostridium sensu stricto1, Faecalibacterium, Fusicatenibacter, Haemophilus, Prevotella9, Ruminococcaceae NK4A214 group, Sellimonas, and an unknown genus. In addition, the Mollicutes RF9 and Selenomonadales orders were also identified.

These findings imply an elevated risk of OP onset. Comprehensive results are presented in Tables 2, 3, 4, and Figure 3. While not attaining statistical significance after FDR correction (q > 0.1), we posit that these outcomes suggest a potential association, meriting further investigation in forthcoming studies.

### 3.3. Heterogeneity and sensitivity analysis

**Figure 4.**
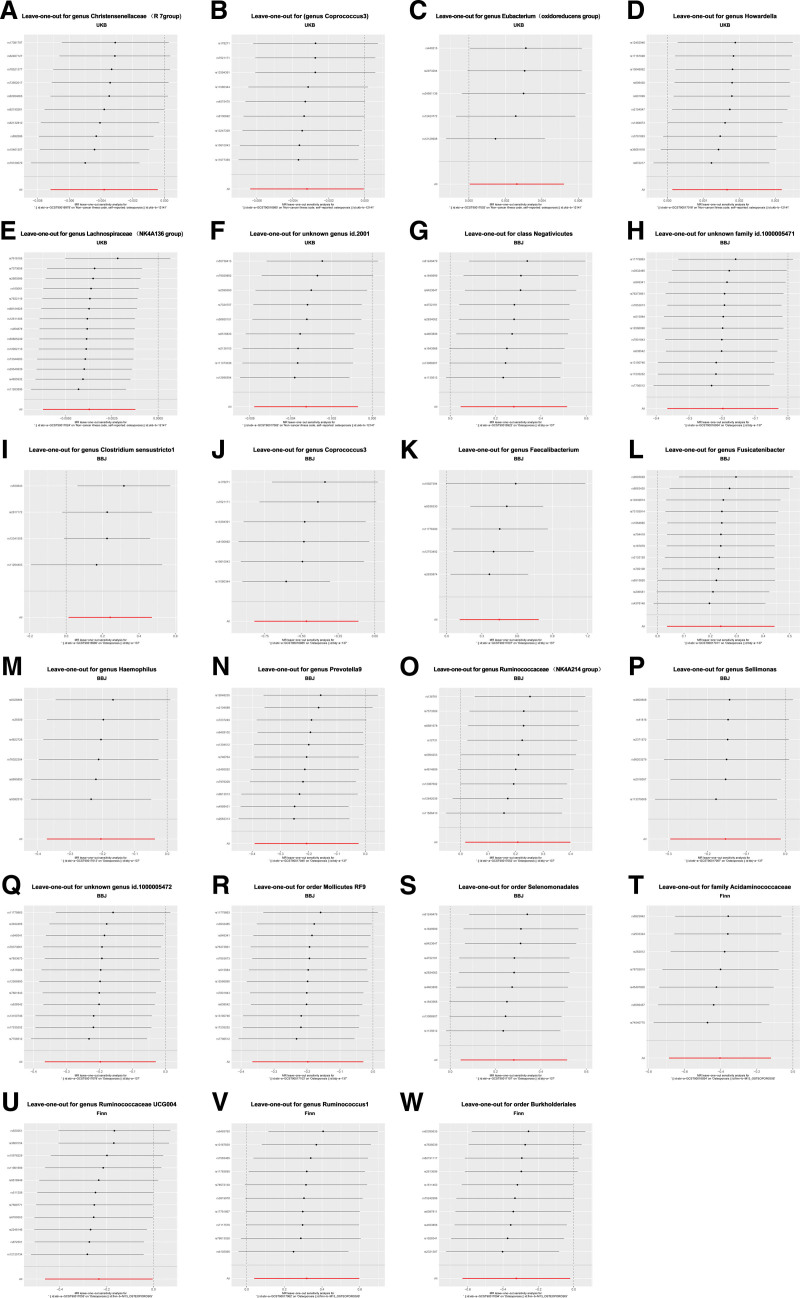
Leave-one-out analysis of the causal relationship between gut microbiota and osteoporosis.

Within these 23 causal associations, the IVs demonstrated F-statistics ranging from 17.28 to 29.33, effectively mitigating biases arising from weak IVs. Cochran IVW Q-test revealed no significant heterogeneity among these IVs. No indication of heterogeneity emerged in the genetic variations of the GM (refer to the funnel plot in the table). None of the MR-Egger regression intercepts deviated from zero, signifying the absence of directional horizontal pleiotropy influence (all intercept *P* value > .05). Furthermore, the leave-one-out analysis unveiled no substantial deviations in the causal relationship between GM and OP, indicating that no single genetic variant propels any identified causal association, as depicted in Figure 4.

## 4. Discussion

OP, a global health concern, has garnered considerable attention. Despite extensive research efforts over the years, its precise etiology remains elusive. With the recent advent of microbiomics, researchers have shifted their focus toward understanding the intricate interplay between GM and various diseases, including OP.^[10,25,26]^

The intricate relationship between GM and the health and diseases of their hosts has emerged as a focal point of scientific inquiry. Preliminary studies have unveiled the pivotal role of GM in skeletal health.^[8,27]^ These microorganisms not only facilitate nutrient absorption^[28]^ but also engage in profound interactions with the host immune system,^[29,30]^ thereby influencing inflammation and bone metabolism.^[31,32]^ For instance, foundational research has identified that GM can impact bone health through the modulation of inflammatory responses,^[33]^ influence calcium uptake,^[31,34]^ and interference with signaling pathways associated with bone metabolism.^[35]^

In this study, we applied MR analysis, a robust statistical approach, to delve into potential causal relationships. Leveraging publicly available GWAS summary data, we embarked on a comprehensive exploration of the connection between GM and OP. Notably, our findings bolster the perspective of a definitive causal link between GM and OP. Research by Wei et al has identified associations between OP risk and the Bacteroidetes phylum, Bacteroides, Lactobacillus phylum, Escherichia, and Eggerthella genera.^[36]^ The Bacteroidetes phylum comprises various Gram-negative bacteria in the gastrointestinal tract, including the Bacteroides genus,^[37]^ aligning well with our findings. In contrast to these earlier findings, our study uniquely illuminates the significant, inverse association between the genus Coprococcus3 and OP risk. This focus on Coprococcus3 represents a novel contribution, emphasizing its critical role in potentially mitigating OP risk and distinguishing our research from the study conducted by Zeng et al.^[38]^ These revelations not only provide novel insights into the pathogenesis of OP but also pave the way for innovative preventative and therapeutic strategies. For instance, modulating the composition of GM might mitigate OP risk. Furthermore, these insights offer potential new biomarkers for early OP diagnosis and risk assessment.

While this study reveals the causal relationship between GM and OP through MR analysis, it does come with certain limitations. Firstly, although MR can help mitigate the impact of confounding variables, our reliance on summary statistics rather than raw data, along with the lowest taxonomic level in the exposure dataset being the genus, restricts our ability to delve into the causal link at the species level between GM and OP. The SNPs employed in the analysis did not meet the conventional Genome-Wide Association Study (GWAS) significance threshold (*P* < 5 × 10^−8^). Secondly, the sample used in this study originates from a specific database, potentially introducing geographical and population biases, thereby necessitating further validation for broader applicability. Lastly, the composition of GM is influenced by a multitude of factors, including dietary habits, lifestyle, and medication, which were not exhaustively accounted for in this research. Our emphasis on the genus Coprococcus3 as a novel insight into the GM role in OP prevention distinctly advances our understanding beyond current literature, setting a foundation for future research to build upon.

## 5. Conclusion

In conclusion, this study offers novel insights into the role of GM in the pathogenesis of OP. Subsequent research is essential to further elucidate this relationship, delineate the mechanisms through which GM affects skeletal health, and leverage this knowledge for the prevention and treatment of OP.

## Author contributions

**Conceptualization:** Jun Ma, Chong-Yi Sun.

**Data curation:** Jun Ma, Xin-Ran Wang, Yu-Xin Zhou, Wei-Jin Zhou, Jian-Nan Zhang, Chong-Yi Sun.

**Formal analysis:** Jun Ma, Xin-Ran Wang, Yu-Xin Zhou, Wei-Jin Zhou, Jian-Nan Zhang, Chong-Yi Sun.

**Writing – original draft:** Jun Ma, Xin-Ran Wang.

**Writing – review & editing:** Chong-Yi Sun.

## References

[1] Yang Z Xing D Dong Z. Epidemiological investigation of 387 individuals over 65 years old with osteoporotic fractures. Altern Ther Health Med. 2023;29:207–11. 36735718

[2] Salari N Darvishi N Bartina Y. Global prevalence of osteoporosis among the world older adults: a comprehensive systematic review and meta-analysis. J Orthop Surg Res. 2021;16:669. 34774085 10.1186/s13018-021-02821-8 PMC8590304

[3] LeBoff MS Greenspan SL Insogna KL. The clinician’s guide to prevention and treatment of osteoporosis. Osteoporos Int. 2022;33:2049–102. 35478046 10.1007/s00198-021-05900-y PMC9546973

[4] Kalinkovich A Livshits G. Biased and allosteric modulation of bone cell-expressing G protein-coupled receptors as a novel approach to osteoporosis therapy. Pharmacol Res. 2021;171:105794. 34329703 10.1016/j.phrs.2021.105794

[5] Spigoni V Cinquegrani G Iannozzi NT. Activation of G protein-coupled receptors by ketone bodies: clinical implication of the ketogenic diet in metabolic disorders. Front Endocrinol (Lausanne). 2022;13:972890. 36339405 10.3389/fendo.2022.972890 PMC9631778

[6] Kumar J Rani K Datt C. Molecular link between dietary fibre, gut microbiota and health. Mol Biol Rep. 2020;47:6229–37. 32623619 10.1007/s11033-020-05611-3

[7] D’Amelio P Sassi F. Gut Microbiota, immune system, and bone. Calcif Tissue Int. 2018;102:415–25. 28965190 10.1007/s00223-017-0331-y

[8] Lu L Chen X Liu Y. Gut microbiota and bone metabolism. FASEB J. 2021;35:e21740. 34143911 10.1096/fj.202100451R

[9] Li D Liu Y Yang X. The role of probiotics and prebiotics in osteoclastogenesis and immune relevance. Curr Med Chem. 2021;28:5228–47. 33726643 10.2174/0929867328666210316115126

[10] Huang R Liu P Bai Y. Changes in the gut microbiota of osteoporosis patients based on 16S rRNA gene sequencing: a systematic review and meta-analysis. J Zhejiang Univ Sci B. 2022;23:1002–13. 36518053 10.1631/jzus.B2200344 PMC9758719

[11] Seely KD Kotelko CA Douglas H. The human gut microbiota: a key mediator of osteoporosis and osteogenesis. Int J Mol Sci . 2021;22:9452. 34502371 10.3390/ijms22179452 PMC8431678

[12] Zemanova N Omelka R Mondockova V. Roles of gut microbiome in bone homeostasis and its relationship with bone-related diseases. Biology (Basel). 2022;11:1402. 36290306 10.3390/biology11101402 PMC9598716

[13] Bowden J Holmes MV. Meta-analysis and Mendelian randomization: a review. Res Synth Methods. 2019;10:486–96. 30861319 10.1002/jrsm.1346 PMC6973275

[14] Kurilshikov A Medina-Gomez C Bacigalupe R. Large-scale association analyses identify host factors influencing human gut microbiome composition. Nat Genet. 2021;53:156–65. 33462485 10.1038/s41588-020-00763-1 PMC8515199

[15] Lotta LA Kettunen J Ahola-Olli AV. FinnGen: Unique genetic insights from combining isolated population and national health register data. Nature. 2023;613:64–73.

[16] Okbay A Wu Y Wang N. Polygenic prediction of educational attainment within and between families from genome-wide association analyses in 3 million individuals. Nat Genet. 2022;54:437–49. 35361970 10.1038/s41588-022-01016-z PMC9005349

[17] Sanna S van Zuydam NR Mahajan A. Causal relationships among the gut microbiome, short-chain fatty acids and metabolic diseases. Nat Genet. 2019;51:600–5. 30778224 10.1038/s41588-019-0350-x PMC6441384

[18] Burgess S Dudbridge F Thompson SG. Combining information on multiple instrumental variables in Mendelian randomization: comparison of allele score and summarized data methods. Stat Med. 2016;35:1880–906. 26661904 10.1002/sim.6835 PMC4832315

[19] Bowden J Davey Smith G Burgess S. Mendelian randomization with invalid instruments: effect estimation and bias detection through Egger regression. Int J Epidemiol. 2015;44:512–25. 26050253 10.1093/ije/dyv080 PMC4469799

[20] Hartwig FP Davey Smith G Bowden J. Robust inference in summary data Mendelian randomization via the zero modal pleiotropy assumption. Int J Epidemiol. 2017;46:1985–98. 29040600 10.1093/ije/dyx102 PMC5837715

[21] Geroldinger A Lusa L Nold M. Leave-one-out cross-validation, penalization, and differential bias of some prediction model performance measures-a simulation study. Diagn Progn Res. 2023;7:9. 37127679 10.1186/s41512-023-00146-0 PMC10152625

[22] Storey JD Tibshirani R. Statistical significance for genomewide studies. Proc Natl Acad Sci U S A. 2003;100:9440–5. 12883005 10.1073/pnas.1530509100 PMC170937

[23] Hemani G Tilling K Davey Smith G. Orienting the causal relationship between imprecisely measured traits using GWAS summary data. PLoS Genet. 2017;13:e1007081. 29149188 10.1371/journal.pgen.1007081 PMC5711033

[24] Verbanck M Chen CY Neale B. Detection of widespread horizontal pleiotropy in causal relationships inferred from Mendelian randomization between complex traits and diseases. Nat Genet. 2018;50:693–8. 29686387 10.1038/s41588-018-0099-7 PMC6083837

[25] Yatsonsky Ii D Pan K Shendge VB. Linkage of microbiota and osteoporosis: a mini literature review. World J Orthop. 2019;10:123–7. 30918795 10.5312/wjo.v10.i3.123 PMC6428997

[26] Hao ML Wang GY Zuo XQ. Gut microbiota: an overlooked factor that plays a significant role in osteoporosis. J Int Med Res. 2019;47:4095–103. 31436117 10.1177/0300060519860027 PMC6753565

[27] Cronin O Lanham-New SA Corfe BM. Role of the microbiome in regulating bone metabolism and susceptibility to osteoporosis. Calcif Tissue Int. 2022;110:273–84. 34870723 10.1007/s00223-021-00924-2 PMC8860778

[28] Shin AS Gao X Bohm M. Characterization of proximal small intestinal microbiota in patients with suspected small intestinal bacterial overgrowth: a cross-sectional study. Clin Transl Gastroenterol. 2019;10:e00073. 31464691 10.14309/ctg.0000000000000073 PMC6736222

[29] Scheidt-Nave C Bismar H Leidig-Bruckner G. Serum interleukin 6 is a major predictor of bone loss in women specific to the first decade past menopause. J Clin Endocrinol Metab. 2001;86:2032–42. 11344203 10.1210/jcem.86.5.7445

[30] Sponholtz TR Zhang X Fontes JD. Association between inflammatory biomarkers and bone mineral density in a community-based cohort of men and women. Arthritis Care Res (Hoboken). 2014;66:1233–40. 24375982 10.1002/acr.22270 PMC4069198

[31] Li L Rao S Cheng Y. Microbial osteoporosis: The interplay between the gut microbiota and bones via host metabolism and immunity. Microbiologyopen. 2019;8:e00810. 31001921 10.1002/mbo3.810 PMC6692530

[32] Resta-Lenert S Barrett KE. Probiotics and commensals reverse TNF-alpha- and IFN-gamma-induced dysfunction in human intestinal epithelial cells. Gastroenterology. 2006;130:731–46. 16530515 10.1053/j.gastro.2005.12.015

[33] Sjögren K Engdahl C Henning P. The gut microbiota regulates bone mass in mice. J Bone Miner Res. 2012;27:1357–67. 22407806 10.1002/jbmr.1588 PMC3415623

[34] Wallace TC Marzorati M Spence L. New frontiers in fibers: innovative and emerging research on the gut microbiome and bone health. J Am Coll Nutr. 2017;36:218–22. 28318400 10.1080/07315724.2016.1257961

[35] Grüner N Ortlepp AL Mattner J. Pivotal role of intestinal microbiota and intraluminal metabolites for the maintenance of gut-bone physiology. Int J Mol Sci . 2023;24:5161. 36982235 10.3390/ijms24065161 PMC10048911

[36] Wei M Li C Dai Y. High-throughput absolute quantification sequencing revealed osteoporosis-related gut microbiota alterations in han Chinese elderly. Front Cell Infect Microbiol. 2021;11:630372. 33996619 10.3389/fcimb.2021.630372 PMC8120270

[37] Mishra SS Bapat SK Tewari JP. Preliminary phytochemical and pharmacological study of Symplocos racemosa (Roxb.). Indian J Physiol Pharmacol. 1964;8:181–8. 5831718

[38] Zeng HQ Li G Zhou KX. Causal link between gut microbiota and osteoporosis analyzed via Mendelian randomization. Eur Rev Med Pharmacol Sci. 2024;28:542–55. 38305631 10.26355/eurrev_202401_35052

